# iPSCs from people with MS can differentiate into oligodendrocytes in a homeostatic but not an inflammatory milieu

**DOI:** 10.1371/journal.pone.0233980

**Published:** 2020-06-08

**Authors:** Itzy E. Morales Pantoja, Matthew D. Smith, Labchan Rajbhandari, Linzhao Cheng, Yongxing Gao, Vasiliki Mahairaki, Arun Venkatesan, Peter A. Calabresi, Kathryn C. Fitzgerald, Katharine A. Whartenby

**Affiliations:** 1 Program in Cellular and Molecular Medicine, Johns Hopkins University School of Medicine, Baltimore, Maryland, United States of America; 2 Department of Neurology, Johns Hopkins University, School of Medicine, Baltimore, Maryland, United States of America; 3 Institute for Cell Engineering, Johns Hopkins University School of Medicine, Baltimore, Maryland, United States of America; 4 Solomon H. Snyder Department of Neuroscience, Johns Hopkins University School of Medicine, Baltimore, Maryland, United States of America; 5 Department of Epidemiology Johns Hopkins University School of Medicine, Baltimore, Maryland, United States of America; 6 Department of Oncology, The Sidney Kimmel Comprehensive Cancer Center, Johns Hopkins University School of Medicine, Baltimore, Maryland, United States of America; Instituto Cajal-CSIC, SPAIN

## Abstract

Multiple sclerosis (MS) is an inflammatory and demyelinating disease of the central nervous system (CNS) that results in variable severities of neurodegeneration. The understanding of MS has been limited by the inaccessibility of the affected cells and the lengthy timeframe of disease development. However, recent advances in stem cell technology have facilitated the bypassing of some of these challenges. Towards gaining a greater understanding of the innate potential of stem cells from people with varying degrees of disability, we generated induced pluripotent stem cells (iPSCs) from peripheral blood mononuclear cells derived from stable and progressive MS patients, and then further differentiated them into oligodendrocyte (OL) lineage cells. We analyzed differentiation under both homeostatic and inflammatory conditions via sustained exposure to low-dose interferon gamma (IFNγ), a prominent cytokine in MS. We found that all iPSC lines differentiated into mature myelinating OLs, but chronic exposure to IFNγ dramatically inhibited differentiation in both MS groups, particularly if exposure was initiated during the pre-progenitor stage. Low-dose IFNγ was not toxic but led to an early upregulation of interferon response genes in OPCs followed by an apparent redirection in lineage commitment from OL to a neuron-like phenotype in a significant portion of the treated cells. Our results reveal that a chronic low-grade inflammatory environment may have profound effects on the efficacy of regenerative therapies.

## Introduction

Multiple sclerosis (MS) is an inflammatory demyelinating disease of the central nervous system (CNS) that has become the most common cause of progressive neurological disability in young adults. While the etiology of this disease remains unknown, data suggest that it likely involves a combination of genetic, immunological, and environmental factors, all of which may influence pathology, symptomatic presentation, and disease course and outcome [1]. Despite having been characterized more than 100 years ago, its pathophysiology remains elusive [2], and both the clinical course and disease outcome are highly variable [3, 4].

Many disease-modifying therapies have been developed to combat disability, some of which have significantly improved the course of MS, but have thus far been unable to stop or prevent neurodegeneration in its progressive phase [5]. One feature of MS that has recently come into focus for new therapeutics is the potential to repair demyelination. Despite the fact that the adult CNS has an available pool of oligodendrocyte progenitor cells (OPCs), and a single oligodendrocyte (OL) has the capacity to produce 40 myelin segments [6], remyelination in patients with MS is still incomplete. One study showed that only approximately 20% of patients are thought to remyelinate to some extent [3], but the mechanisms separating successful and failed remyelination are not well known [7], even when the progenitors of myelin-producing cells are present at the sites of injury [8, 9]. For OPCs to contribute to remyelination, they likely must migrate to the sites of injury, proliferate, and differentiate into OLs [10]. Each of these processes can be inhibited by cytokines (e.g., IL-6, IL-17, osteopontin, IFNγ, TNFα), chemokines (e.g., CXCL1, CXCL2, CXCL10, CXCL11), cytolytic proteins (e.g., lymphotoxin-a and perforin), and signaling factors (e.g., astrocyte-derived endothelin-1 (ET-1), all of which are known to be present at demyelinated areas [11–16], thereby establishing a potentially challenging environment for repair.

MS is notably heterogeneous but can be broadly categorized into two subtypes, relapsing remitting and progressive MS, depending on the presentation and course [1,4]. Within both subtypes is a wide range of disease severity, with some people exhibiting a stable course with limited or no disability, while others quickly decline, often with rapid accumulation of severe disability. The mechanisms responsible for these varying outcomes are unclear, and whether there are innate differences in the ability to repair remain to be fully understood. We thus sought to evaluate the potential of iPSCs from people with varying degrees of disability to differentiate into OLs, and to determine mechanisms that might contribute to failure to differentiate into myelinating oligodendrocytes. Thus, we established a platform to generate and investigate OL lineage cells by employing a relatively recent stem cell technology that allows de-differentiation of peripheral blood mononuclear cells (PBMCs) from adult donors into iPSCs which can then be followed by differentiation into multiple cells of interest, including OPCs [17–20]. Using this system, cells from multiple patients can be simultaneously generated.

The ability to study human cells, particularly from people with disease, adds an important element to the study of MS. While much information on OPC biology and differentiation has been generated in rodent developmental systems that provide a framework for complementary human studies, some critical notable differences may be relevant to human disease. For example, most rodent studies utilize OPCs from neonatal animals during a developmental state, and OPCs from adults exhibit at least some different properties [21–23]. Rodent cells differentiate in a matter of days, while human cells require months in culture, thereby allowing a time scale that is much more reflective of humans and allows chronic exposure to varying conditions. Further, rodent cells are typically obtained from healthy pups, which may not be reflective of disease states, and are from homogeneous donors. We thus developed an *in vitro* platform in which iPSCs were generated from PBMCs of people with either stable MS with low disability score or progressive MS with high disability score. These cells were then differentiated into mature OLs. During the course of culture, the cells were analyzed both in a non- inflammatory/homeostatic state and in an inflammatory environment to better reflect a disease milieu. Specifically, to model an inflammatory environment, we exposed developing OPCs to interferon gamma (IFNγ), which is a cytokine of particularly high relevance in MS. IFNγ is secreted by T cells, natural killer cells, and myeloid lineage cells and is often present in MS lesions [24, 25]. Rodent studies in both *in vivo* and *in vitro* settings have demonstrated that in addition to its pro-inflammatory properties, IFNγ also inhibited the differentiation of OPCs into mature OLs, caused cell death through multiple pathways [26–28], and affected remyelination through endoplasmic reticulum stress [29]. The net result of these combined effects likely promotes a hostile environment for remyelination.

In support of these hypotheses, the results of our present studies show that iPSCs from all MS patients were able to differentiate into mature, myelinating OLs but that chronic exposure to low-dose IFNγ early in development had a profound negative effect on differentiation. These results demonstrate the ability of iPSCs from heterogeneous states of disease to differentiate into OLs in the absence of inflammation. Our findings also illustrate the implications of long-term exposure to even very low concentrations of IFNγ, and highlight the importance of evaluating both cytoprotective and remyelination-promoting agents in the context of an inflammatory microenvironment that may influence repair in people with MS.

## Materials and methods

### Patients and cells

PBMCs were obtained from donors with full informed consent under a protocol approved by the Johns Hopkins Medicine Institutional Review Board. Patients were evaluated in the MS Clinic and categorized for disease severity based on their expanded disability status scale (EDSS) score and disease duration. Patients were selected for age, sex, and duration of disease greater than 20 years but to be discordant for EDSS low (<3.0) or EDSS high (≥6.5). Three stable, three progressive and four healthy controls were used in these studies. PBMCs were isolated via Ficoll and stored in liquid nitrogen until use.

### Reprograming and characterization of human iPSCs

Cryopreserved PBMCs were thawed for culture and reprogrammed to iPSCs via transduction of episomal vectors containing Oct3/4, Sox2, Klf4 c-Myc, and Bcl-xl factors [30–32]. Generated iPSC lines were cultured and expanded in E8 medium with recombinant human vitronectin proteins [32, 33]. The iPSCs were characterized by cytology, karyotype analysis, and flow cytometry analysis as previously described [30, 31]. Cell colonies after reprogramming displayed human iPS cell morphology and abundant expression of TRA-1-60, a commonly used marker of undifferentiated pluripotent human stem cells. Generated iPSC lines also exhibited normal karyotype by standard G-banding (300–500 bands) karyotyping method [34–36].

### Differentiation of iPSCs-derived OPCs and OLs

Human iPCS were differentiated to OPCs and OLs as previously described [37], with minor modifications of cell density.

### Cell sorting

Cells were sorted based on expression of either PDGFRα or O4, depending on the experiment and maturation state of the cells. For PDGFRα expression, cells were sorted after 56 to 60 days of culture. For O4 expression, cells were sorted at approximately day 75. Cells were dissociated to a single cell suspension with TrypLE, filtered, Fc blocked with True Stain FcX (1:20, Cat # 422302, BioLegend), then stained with either PDFGRα/CD140a (1:10 Cat# 323506, BioLegend) or O4 (1:50 Cat # 130-117-357, Milteny), and cell density was adjusted for sorting. Sorted cells were placed in culture to recover and then treated as described for each experimental technique.

### Immunofluorescence (IF) characterization

After sorting, cells were cultured until analysis and then were fixed with cold 4% paraformaldehyde for 20 minutes, permeabilized with 0.2% TX-100 in 1X PBS for 20 minutes, blocked with 3% NGS and 0.1% TX in 1X PBS for 1 hour. MBP primary antibody was used at 1:500 dilution in blocking buffer and incubated at 4°C overnight, followed by washing and incubation with secondary goat anti-mouse Alexa Fluor^®^ 488 (1:500) for 2 hours in blocking buffer. Nuclear staining was conducted with 1μM DAPI, incubated for 20 minutes in PBS. All steps with the exception of the primary antibody were performed at room temperature.

### Cytokine treatment

Bulk OPC cultures were treated with recombinant human IFNγ (PeproTech) at a dose of 10 ng/ml from day 36 to day 82–85 or from day 60 to day 82–85, with a change of medium every 48 hours.

### Flow cytometry analysis

Cells were prepared as described above, followed by staining with antibodies for cell surface markers, PDFGRα/CD140a (1:10, Cat #323506, BioLegend), O4 (1:50, Cat # 130-117-357, Miltenyi). Viability was determined by a Live/Dead Staining Kit (1:500,Cat #L34966,ThermoFisher) For intracellular staining, cells were fixed and permeabilized with the Foxp3 transcription factor staining buffer kit (Cat # 00-5523-00, eBioscience) according to the manufacturer’s protocol, followed by staining of the intracellular marker Olig2 (1:50, Cat # MABN50A4, EMD Millipore).

### Co-culture of neurons and OPCs

Cultured cells were harvested at day 84 and were seeded in one of two compartmentalized chambers, which are separated by micro-channels as previously described [38] at a density of 10,000 cells per chamber. The second chamber was seeded with neurons, generated from hESC (H9 cell line), which allows the axons from the neurons to protrude through the micro-tunnels, providing a substrate for the myelinating OLs. Approximately 30 days later, cells were fixed with ice cold 4% paraformaldehyde for 20 minutes followed by washing with PBS. Cells were then blocked with 3% normal goat serum and 0.1% Triton-X in PBS for 1 hour, and stained with primary antibodies of mouse SMI99 MBP (1:50, Cat#SMI-99P, Covance), or chicken neurofilament (NF) at 1:200, (Cat# NF-M, Aves Lab Inc), overnight at 4°C. Samples were washed three times after incubation with primary antibody with PBS, and then appropriate secondary antibodies conjugated with Alexa Fluor (mouse, chicken or rabbit, all at 1:250, Invitrogen) were incubated for 1 hour and 30 minutes at room temperature. Cells were incubated for 5 minutes with 1μM DAPI (4′, 6-diamidino-2′- phenylindoldihydrochloride; Invitrogen) as a nuclear counter-stain. Zeiss live-cell inverted microscope (Axio Observer; Zeiss, Germany) was used for imaging at 20X magnification.

### Quantitative PCR

RNA was isolated via an RNeasy kit (Qiagen) followed by cDNA preparation with an iScript cDNA Synthesis Kit (Bio-Rad). Reactions were conducted with iQ SYBR Green Super Mix (BIO-RAD) with primers manufactured by Integrated DNA Technologies (Coralville, IA, USA). Primer sequences were: Human GAPDH (Forward 5’-AAG GTG AAG GTC GGA GTC AAC-3’, Reverse 5’- GGG GTC ATT GAT GGC AAC AAT A-3’), Human MBP (Forward 5’-CTA TAA ATC GGC TCA CAA GG-3’, Reverse 5’-AGG CGG TTA TAT TAA GAA GC-3’).

### RNA sequencing

OPC cultures were FACS-sorted for expression of PDGFRα as above, re-plated and cultured with or without the addition of 10 ng/ml of IFNγ for 16 hours, followed by RNA isolation (RNeasy Plus mini kit, Qiagen). Libraries were prepared using the Illumina TruSeq Stranded Total RNA LT Sample Prep and were then sequenced on an Illumina HS2500 and Novaseq6000.

### RNA-seq analysis

Transcript counts were quantified by pseudo-alignment against the human transcriptome (GRCh38.p13) using Salmon v1.1.0 [39], after filtration of ribosomal RNA transcripts using BBSplit. Raw counts were imported into R v3.6.2 and aggregated to gene level counts with tximport v1.14.0 [40] and then DESeq2 v 1.26.0 [41] was used to normalize, perform exploratory analysis, and test for differential gene expression.

Volcano plots were generated with the Bioconductor package EnhancedVolcano v1.4.0(https://github.com/kevinblighe/EnhancedVolcano). The functional analysis was generated through the use of Ingenuity Pathway Analysis (Qiagen) using thresholds of adjusted p value<0.05 and Log_2_ FC less than -1 or greater than 1 [42]. The shell scripts and R script used to quantify and analyze are available upon request. Raw and processed data are publicly available: (GEO; https:/www./ncbi/nlm/nih/gov/gds) with accession number GSE147315.

### Transmission electron microscopy (TEM)

Cells were fixed in 2.5% glutaraldehyde, 3mM MgCl2 in 0.1 M sodium cacodylate buffer, pH 7.2 for one hour at room temperature. Samples were then rinsed and postfixed in 1% osmium tetroxide, 0.8% potassium ferrocyanide in 0.1 M sodium cacodylate for 1–2 hours on ice in the dark. The samples were then rinsed in 100 mM maleate buffer, followed by incubation with uranyl acetate (2%) in 100 mM maleate for 1 hr in the dark and then were dehydrated in a graded series of ethanol concentrations, and embedded in Eponate 12 (Ted Pella), then polymerized at 37°C for 2–3 days followed by incubation at 60°C overnight. Thin sections, 60 to 90 nm, were cut with a diamond knife on the Reichert- Jung Ultracut E ultramicrotome and picked up with 2x1 mm formvar copper slot grids. Grids were stained with 2% uranyl acetate in 50% methanol followed by lead citrate and observed with a Philips CM120 TEM at 80 kV. Images were captured with an AMT CCD XR80 (8 megapixel camera—side mount AMT XR80 –high-resolution high-speed camera).

### Statistical analysis

A statistical analysis was conducted on the percentages of cells expressing O4 or Olig2 between and among controls and MS patients (categorized as stable or progressive) with and without IFNγ treatment (untreated, at day 36 and at day 60) using generalized estimating equations (GEE) to account for inclusion of multiple samples per patient. Statistical tests were two-sided, and significance was determined using a p-value less than 0.05. All analyses were performed using R (version 3.10; www.R-project.org).

## Results

### iPSCs successfully differentiated to mature OLs

**Fig 1:**
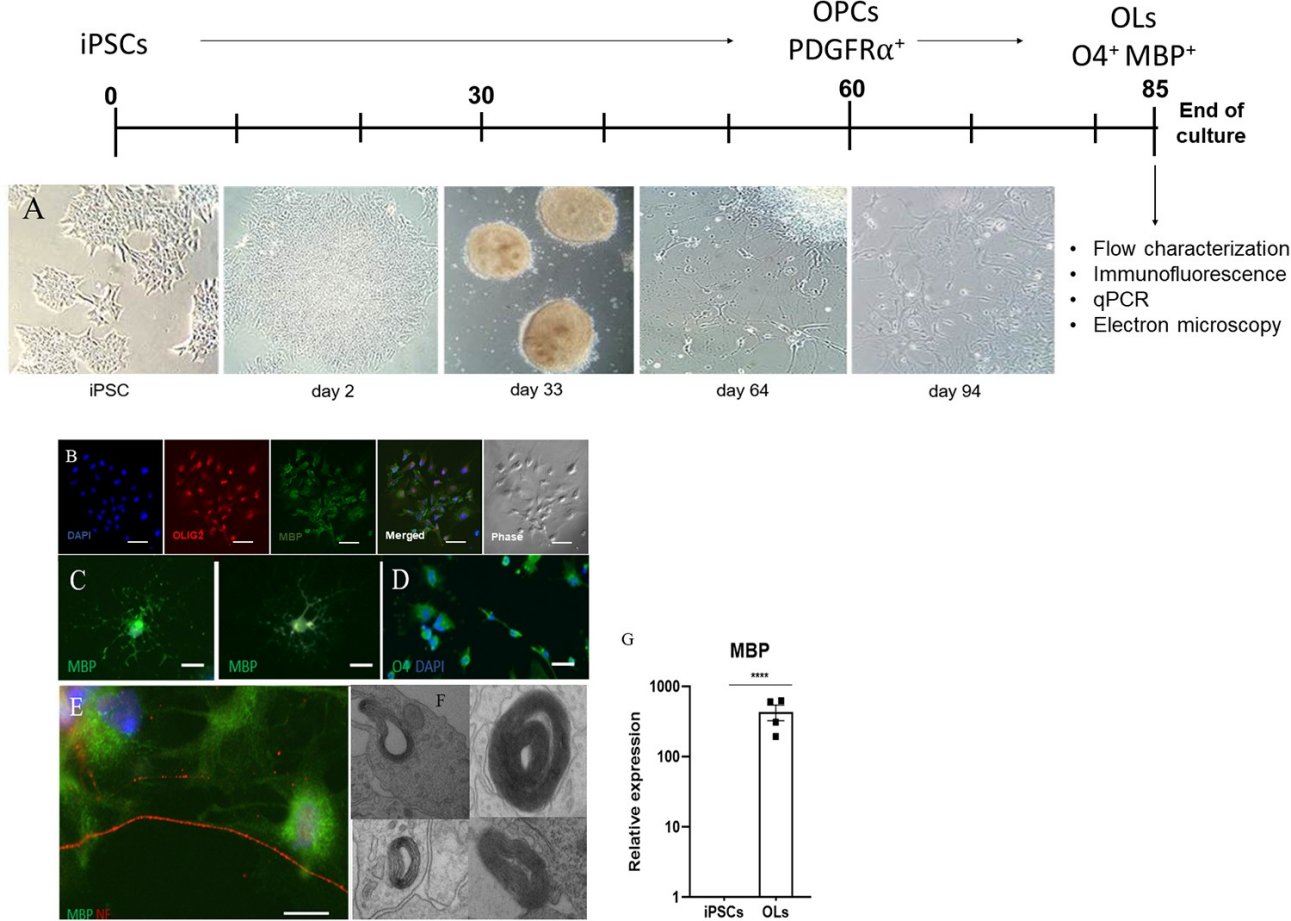
Characterization of MS iPSC-derived OLs. Validation of OL lineage identity was conducted during the course of the differentiation via multiple techniques. **A** shows a phase contrast analysis demonstrating the morphological changes during the progression of OL development. For analysis of purified populations, cells were FACS-sorted at two different time points. For **B and C**, cells were FACS-sorted for PDGFRα expression at the OPC stage after 60 days of differentiation and analyzed after day 85 by immunofluorescence for expression of canonical markers. Cells were identified with the nuclear DAPI stain and labeled with antibodies for Olig2 and MBP as shown, with a merge in **B**. A selected image showing myelin basic protein (MBP) staining with characteristic OL morphology is highlighted in **C. D** shows the immunostaining of cells that were FACS-sorted for the expression of O4 at day 75 of culture, then re-cultured for an additional 9 days prior to analysis. As shown, O4 expression remained and was confirmed at day 84 of differentiation. To demonstrate functional myelination, two different approaches were undertaken. First, OLs at day 84 of differentiation from mixed cultures were co-cultured in a chamber format in which neurons remain on one side, but axons project through micro-tunnels to the other side where OLs are cultured. After three weeks of co-culture, cells were immunostained for MBP in green, and neurofilament in red. **E** shows OLs contacting axons, with the appearance of wrapping. The second approach to validate the functional wrapping was transmission electron microscopy (TEM) analysis on OLs from mixed cultures as shown in **F**. As further confirmation of the cells’ function, qPCR analysis shows high levels of expression of MBP compared to iPSCs as shown in **G** (4 cell lines in duplicate, normalized to GAPDH). All images are representative samples encompassing all cell lines. Images 1A-E were taken at a magnification of 20X (scale bar = 50 μm).

PBMC-derived iPSCs were validated as described in methods and then cultured under a standard protocol to generate OL lineage cells. The progression of differentiation occurs over a lengthy time period of 80–90 days for full maturation. This process is shown in a sample phase contrast, which demonstrates the morphologic change associated with differentiation to oligo lineage cells in Fig 1A. To more definitively validate the identity of the oligo lineage cells, we intensively characterized them using a variety of complementary methods. Throughout the culture period, we monitored cells for expression of canonical markers, transcription factors, and ultimately, the critical evidence of OL lineage, namely production of myelin and functional wrapping. Specifically, we characterized the phenotype of our cultures at distinct time points (OPCs, immature and mature OLs), to monitor development. Towards the end of cultures (approximately days 80–85) as cells are reaching maturity, we analyzed cells for the expression of characteristic markers. As Olig2 is a critical transcription factor for the development of OLs, and MBP is the hallmark of a mature OL, we analyzed these in depth. As shown in Fig 1B, cells express both of these markers. OLs exhibit a characteristic morphology in their mature state, and Fig 1C shows highlighted OLs with both this morphology and a concomitant expression of MBP. These figures help to confirm the identity of the cells. To validate the expression of O4, cultures were first sorted by FACS based on the expression of O4 and placed them in culture to mature fully. After culture, cells were then immunostained with O4. As shown in Fig 1D, the cells retained expression through maturity, validating both the expression of the marker and the antibodies used for analysis. The critical function and hallmark of OLs is the production of myelin and wrapping of axons. We therefore assessed this process in different formats. To assess myelination of axons, OL cultures were replated in a split chamber system, in which the OLs were plated in one chamber and neurons were cultured in a separate chamber, while their axons are allowed to migrate through microtunnels into the OL chamber. After approximately 30 days of co-culture, the chambers were subjected to immunofluorescence (IF) analysis with antibodies to MBP and neurofilament (NF). As shown in Fig 1E, mature OLs were able to contact and apparently ensheathe axons. To conduct an ultimate identification of wrapped axons, we employed electron microscopy to reveal the structure. Fig 1F shows representative images of myelinated axons with compact lamellar myelin. Similar results were obtained from all donors. As a further confirmation of the identity of the cells, we conducted qPCR analysis for MBP expression, which provides a specific genetic confirmation of expression of this hallmark molecule. As shown in Fig 1G, OLs had a high level of expression of MBP. Taken together, these data show that iPSCs from MS patients differentiated into OPCs, which then could be further differentiated to functionally mature OLs.

### iPSCs from all donors differentiated into OLs

**Fig 2:**
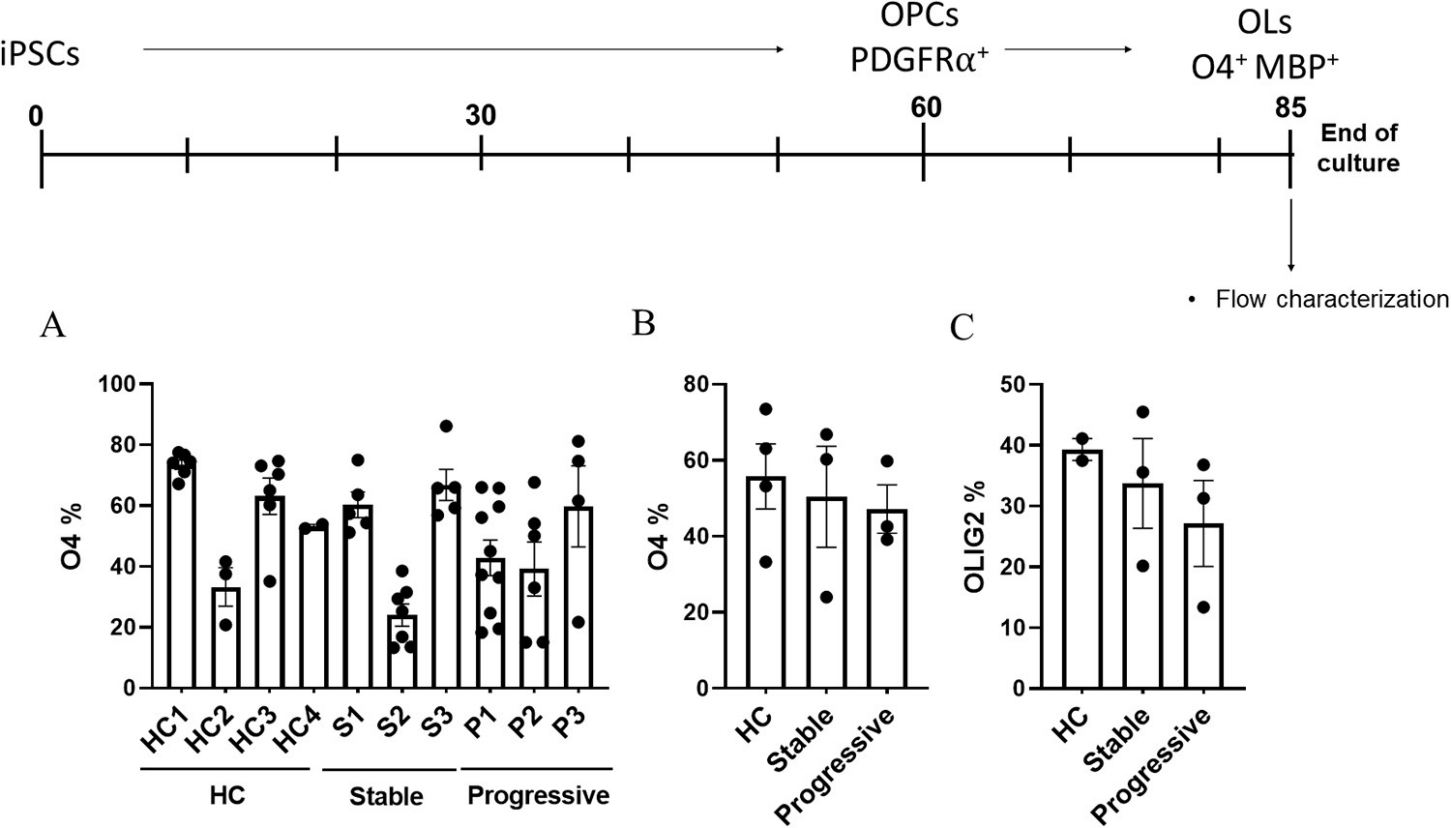
iPSC lines from all donors differentiate to OLs. To quantify and compare the differentiation of iPSCs into OL lineage cells from multiple donors in a more high-throughput manner, flow cytometry analysis was employed. Based on the validation in Fig 1, percentages of cells expressing O4 and Olig2 were determined in cells from mixed cultures at approximately day 85 of differentiation. **A** shows the percentage of O4 expression for each of the four healthy controls (HC1-4), and MS donors classified as stable (S1-3), and progressive (P1-3). Each dot represents one experiment per donor. Number of experiments per donor: HC1 = 6, HC2 = 3, HC3 = 6, HC4 = 2; S1 = 5, S2 = 7, S3 = 5; P1 = 10, P2 = 6, P3 = 4. **B** shows the compilation of the data in A, with the samples from each group combined and averaged. As in A, the data are comprised of four healthy controls, three stable, and three progressive donors. Each dot represents the average for one donor. **C** shows the compilation of the data of expression of Olig2 per group, with each dot representing one patient (not all samples were analyzed for Olig2). Number of donors per group: HC = 2, S = 3; P = 3, with 3–6 experiments per group. Significance was determined with generalized estimating equations with R, and using a p-value less than 0.05.

Having validated the experimental process and identity of the cells, we next conducted a more high-throughput and quantitatively comparable analysis of the generation of OLs from iPSCs. To this end, we cultured and differentiated iPSCs from the six patients and four healthy controls, for approximately 85 days to a mature OL stage, and then profiled large populations by flow cytometry using O4 and Olig2 as identifying markers. O4 has been used to identify and characterize oligo lineage cells from tissue of healthy individuals and from chronic lesions of MS patients [43, 44]. All MS iPSC lines expressed O4, albeit to different extents, which were similar to those reported previously [20]. The results of these analyses showed effective differentiation of all samples, with a broad range of efficiency of differentiation and maturation within (upon repeat culture) and across individuals. Fig 2A shows the efficiency of differentiation for each donor. Some cell lines demonstrated higher degrees of variability than others, but neither variability nor final efficiency of maturation was statistically linked with one cohort. In order to compare among groups, we analyzed the averages of each group, with donors from each cohort combined (Fig 2B). While there was a trend towards lower efficiency in the progressive group compared to healthy controls, there was no significant difference in the aggregates. We similarly analyzed the comparison of Olig2 expression, with similar results (Fig 2C), in which a trend towards a decrease in progressive samples was present, but it did not reach significance. Taken together, these results demonstrated that iPSCs from all donors could ultimately differentiate into mature OLs.

### Chronic exposure to low-dose IFNγ inhibits OPC differentiation

**Fig 3:**
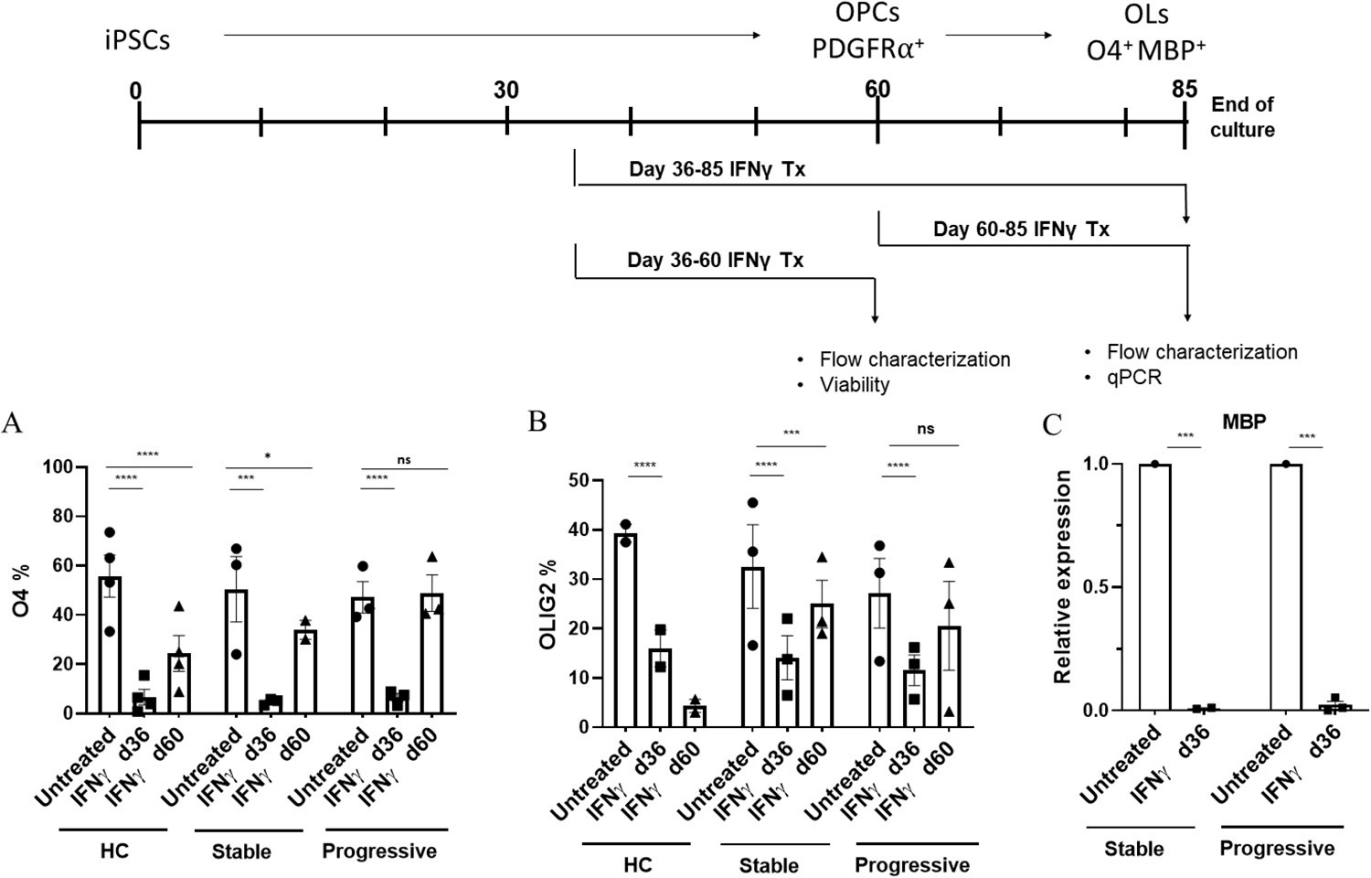
Sustained exposure to low-dose IFNγ inhibits OPC differentiation in MS and healthy control groups. To assess the effects of low-dose (10 ng/mL) IFNγ on OPCs differentiation, mixed cultures were left untreated or treated from either day 36 or 60 to the end of culture, at approximately day 85. Flow cytometry analysis was then conducted to evaluate differentiation. **A** shows the percentage of cells expressing O4 in untreated and IFNγ-treated cultures for each group. Low-dose IFNγ was initiated at either day 36 or 60 as noted. The stable group consists of 3 donors with 3 to 14 experimental replicas, the progressive group contains 3donors with 3 to 17 experimental replicas, and the control group (HC) consists of 4 donors with 11 to 14 experimental replicas, and each dot represents the average of the replicates for each single donor. **B** shows the percentage of cells expressing Olig2 in untreated and IFNγ treated cells in all groups, as in **A**, starting at either day 36 or 60 as indicated. The HC group consists of 2 donors, the stable MS group consists of 3 donors, and the progressive group consists of 3 donors. Each dot represents a single donor, with 3–6 averages/group. **C** shows the results of qPCR analysis of expression of MBP in cultures after the long term (day 36 onward) low-dose IFNγ exposure. At end of culture, samples were processed, and qPCR was conducted for GAPDH and MBP. Shown is a comparison of relative expression of MBP, normalized to GAPDH, revealing further confirmation of the inhibition of OL maturation by the decreased expression of MBP. (2 donors in the stable group, 3 donors in the progressive group, averages of duplicate analysis). Significance was determined with generalized estimating equations with R, and using a p-value less than 0.05.

An important consideration in myelin repair in a chronic disease is that the CNS may not be in a homeostatic state in MS, and that either overt or smoldering inflammation may be present. To investigate the effects of an inflammatory environment on the differentiation of iPSCs into OPCs, we next assessed the process in the presence of sustained exposure to low-dose IFNγ. Parallel cultures of untreated and treated cells were established, with the IFNγ provided during the critical period of development, spanning from day 36 to final analysis, or for some experiments from day 60 to final analysis. At the end of culture, cells were analyzed by flow cytometry for differentiation markers as established in Figs 1 and 2. Fig 3A shows the effects of IFNγ treatment on the differentiation of the donors, separated by groups, with replicates for each donor averaged. As shown in the graphs, IFNγ treatment initiated early during culture profoundly inhibited the development of O4+ OLs in all groups. While iPSCs from some patients differentiated more efficiently than others, the inhibition was uniformly robust, regardless of the nature of disease in the donor. The culture requires a lengthy process, and as cells transition through phases of progenitor plasticity during the course of differentiation, we sought to determine whether IFNγ exposure might produce different effects on cells that were not treated until they had reached the early OPC stage. Interestingly, the inhibitory effects were significantly milder when treatment was delayed to day 60, highlighting a critical period of vulnerability during the early stages (Fig 3A). A similar effect was observed on the expression of Olig2, a transcription factor necessary for activation of the genetic program of OLs (Fig 3B), again supporting the inhibitory effect of IFNγ on the development of mature OLs. As a genetic confirmation of the impact of IFNγ on terminal differentiation, an analysis of MBP expression demonstrated that while untreated cells had high levels of MBP, consistent with mature OL status, those that were exposed to IFNγ had minimal detectable levels, furthering the evidence that OL development was inhibited (Fig 3C). In sum, these data, along with the data from Figs 1 and 2, show that iPSCs from all groups effectively mature to OLs, but that exposure to IFNγ significantly derails this process.

### Sustained exposure to low-dose IFNγ redirects lineage phenotype

**Fig 4:**
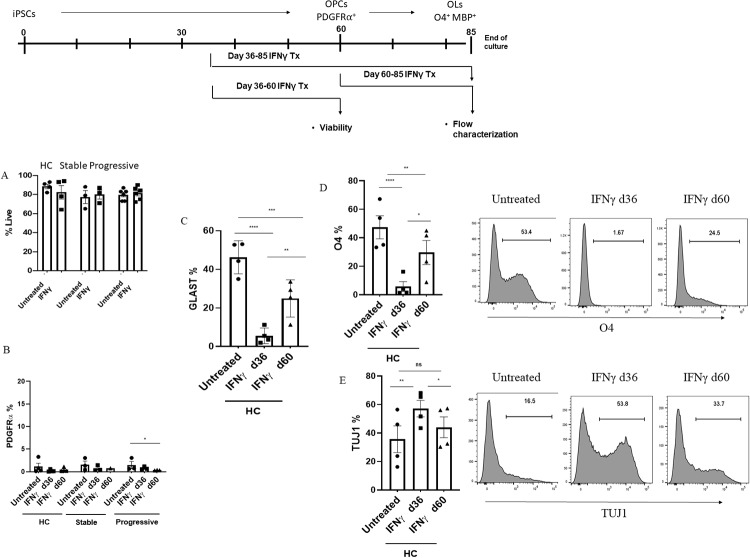
Sustained exposure to low-dose IFNγ appears to redirect differentiation lineage. After approximately 85 days of culture, the identity and viability of the IFNγ-treated cells were determined by flow cytometry. **A** shows the percentage of live cells as determined by flow cytometry viability analysis, of untreated cells and cells treated with IFNγ from day 36 to 60, which is a period of high vulnerability for the cells. Shown are the compiled and averaged results for each group, with each dot representing a single donor, with 1–2 donors per group, repeated 1–4 times. **B** shows the results of flow cytometry analysis for the OPC marker PDGFRα. The percentages of cells in the late-stage culture expressing the marker for each group is shown for both untreated and IFNγ-treated (beginning at either day 36 or 60, as labeled) cells. The HC groups consisted of 4 donors with 2 replicas, the stable group consists of 3 donors, each with 4–7 replicas; the progressive group includes 3 donors with 4–7 replicas of each, and each dot represents one donor. **C** shows the percentages of cells expressing the astrocyte marker GLAST in untreated cells and cells treated with IFNγ, starting at day 36 or 60 (4 HC donors with 1–2 replicas each), and each dot represents one donor. **D** and **E** shows the comparison of the percentages of cells expressing O4 and the neuronal marker βIII tubulin (TUJ1) for untreated and IFNγ-treated cells in the HC group (4 donors, with 2 experimental replicas) starting at day 36 or 60, demonstrating a concomitant increase in TUJ1 to accompany the decrease in O4. Shown are a compilation of data in the graph and representative histograms. Significance was determined with generalized estimating equations with R, and using a p-value less than 0.05.

Consistent with previous studies, we observed no overall increase in cell death in treated cultures, even when cells were exposed to low-dose IFNγ during the high vulnerability period from day 36 to 60 (Fig 4A). Thus, in order to account for the decrease in OLs, we evaluated other populations to identify one with a concomitant increase. Previous rodent studies showed that IFNγ treatment of progenitors led to an increase in PDGFRα progenitors and a decrease in mature OLs by overriding cell cycle exit [27]. We thus quantified PDGFRα expression at the end of the cultures, but did not find a universally similar result in the human cells, regardless of whether treatment started at d36 or d60. As expected at this timepoint, PDGFRα expression was low in untreated cultures, as OPCs have matured into OLs and thus lost expression by this phase. Treatment with IFNγ did not increase the fraction of cells expressing PDGFRα, with the exception of the progressive samples, which while reaching a low level of significance, the percentage of all remained very low (<5%) and thus could not compensate for the loss of the large OL population (Fig 4B). As astrocytes commonly arise from mixed cultures, another possibility was that IFNγ treatment skewed early cells towards an astrocytic profile. To quantify percentages of astrocytes we performed flow cytometry analysis using the GLAST (ACSA-1) marker. Our results showed IFNγ exposure significantly reduced this population as well (Fig 4C).

As development of OL and astrocyte lineages was suppressed, we next investigated the development of neuronal lineages in the presence of IFNγ, via expression of βIII tubulin (TUJ1). In sharp contrast to the suppressive effects on astrocyte and oligo lineages (Fig 4C and 4D), IFNγ exposure led to a concomitant, significant increase in the percentages of cells expressing TUJ1, with similar patterns linked to time of exposure (i.e., from day 36 or day 60) (Fig 4E).

### Short-term IFNγ treatment upregulates immune response pathways

**Fig 5:**
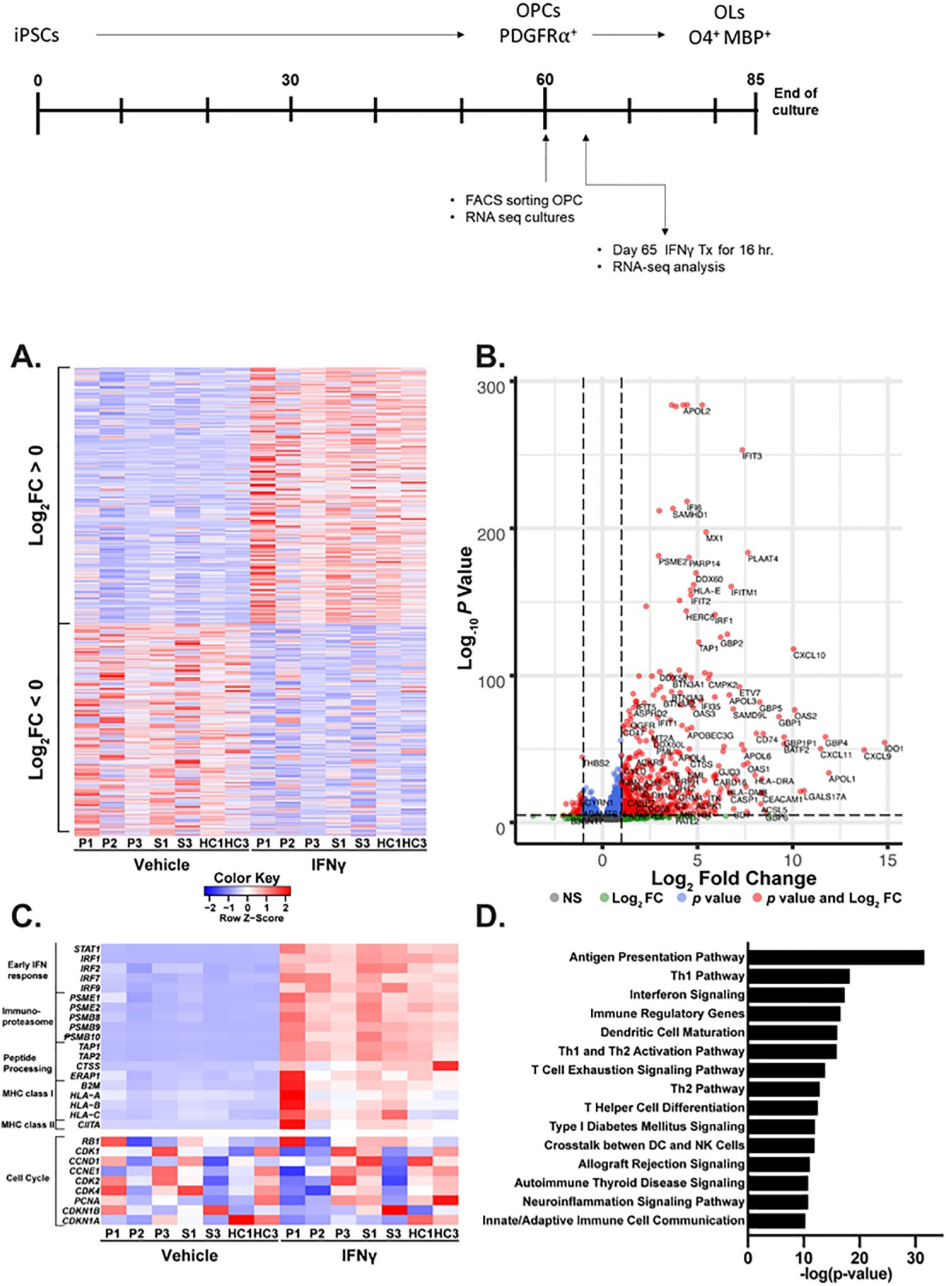
RNA-Seq analysis of IFNγ exposure in PDGFRα sorted OPCs. OPCs were sorted for PDGFRα expression from a total of 7 different cell lines generated from the following donors: 3 people with progressive MS (P1-P3), 2 people with mild/stable MS (S1, S3), and two healthy controls (HC1, HC3). After sorting, cells were plated and exposed to 10ng/μL IFNγ for 16 hours. RNA was then isolated and subjected to RNA-Seq analysis as described in the methods section. **A** shows a heatmap of all differentially expressed genes (defined by having an adjusted p value < 0.05) in two sets: those that are upregulated by IFNγ (Log_2_ Fold Change > 0, n = 1749) on top and those that are downregulated by IFNγ (Log_2_ Fold Change < 0, n = 1453) on the bottom for each of the seven OPCs tested, showing patterns of similarity and differences between groups and individuals. **B** shows a volcano plot with Log_2_ Fold Change on the x-axis and–Log_10_ Adjusted *p* value on the y-axis. Each individual gene is represented by a single dot, and color indicates the following: Red: >2 fold change and adjusted p value < 0.05, Blue: <2 fold change and adjusted p value < 0.05, Green: > 2 fold change and adjusted p value > 0.05, and Grey: < 2 fold change and p value > 0.05. **C** shows a heatmap of IFNγ response genes and immune/antigen presentation genes in the top panel, and a subset of cell cycle control genes previously identified [27]in the bottom panel. **D** shows the results of an Ingenuity Pathway Analysis [42] conducted on genes differentially regulated by IFNγ (Log_2_ Fold Change < -1 or > 1 and adjusted *p* value < 0.05) for canonical pathway enrichment. Top 15 results are shown, sorted by -Log(*p* value).

To better understand the effects of IFNγ treatment on OPC differentiation, we sought to monitor early transcriptional changes through RNA sequencing. To this end, PDGFRα+ cells were sorted at the OPC stage (day 60), allowed to recover in culture, and then treated for 16-hours with low-dose IFNγ. We found a pronounced effect on the transcriptome of OPCs with over 3200 differentially expressed genes; 1749 were upregulated and 1453 downregulated (Fig 5A and 5B). The most induced and highly significant genes were largely those traditionally associated with an inflammatory response such as TAP1, CXCL9, and CD74 (Fig 5B, Table 1). Among the highly upregulated genes, we found early interferon response genes: STAT1, IRF1, IRF2, IRF7, and IRF9, peptide processing genes: TAP1, TAP2, and ERAP1, immunoproteasome component genes: PSME1, PSME2, PSMB8, and PSMB9, antigen presentation genes class I: HLA-A and HLB-B, and class II: CIITA, (Fig 5C, Table 1). Consistent with these findings, functional analysis of differentially regulated genes showed most significant enrichment for antigen presentation and immune activation (Fig 5D). In their rodent studies, Chew et al., 2005 showed that IFNγ kept OPCs in the progenitor state by overriding the cell-cycle exit, which they demonstrated by showing upregulation of cell-cycle proteins on IFNγ-treated cells [27]. Our results did not show a similar differential expression of cell-cycle genes (Fig 5C), indicating that a different mechanism may be operational at this timepoint in human cells. Consistent with suppression of OL development, however, upregulation of DAAM2, which was recently shown to play a role in suppressing OPC differentiation but has not previously been linked with IFNγ signaling in OPCs [45], was observed (Log_2_FC = 1.97, adjusted *p* value = 3.73E-07). Thus, treatment of progenitors at the PDGFRα+ OPC state led to an early, robust upregulation of immune and inflammatory response genes.

**Table 1:** RNA-seq analysis of gene enrichment of IFNγ exposure in PDGFRα sorted OPCs.

Gene	Log_2_ FC	p-adj
IDO1	14.83555	8.17E-53
CXCL9	13.76216	1.18E-47
APOL1	11.90543	2.27E-32
GBP4	11.72463	1.44E-56
CXCL11	11.47197	1.15E-48
LGALS17A	10.60319	1.66E-20
XAF1	10.42001	5.05E-20
OAS2	10.10588	7.54E-75
CXCL10	10.05021	7.80E-116
GBP1P1	9.55261	1.86E-56
BATF2	9.54757	5.92E-52
GBP1	9.28494	3.37E-70
PLA2G2D	8.77572	3.87E-04
GBP6	8.58085	6.02E-07
CIITA	8.44938	1.06E-58
CEACAM1	8.42068	8.08E-19
TMEM140	8.39200	2.36E-28
USP30-AS1	8.33499	1.11E-18
ACSL5	8.33042	8.93E-12
GBP5	8.26802	4.57E-80

Table 1 shows the 20 most upregulated genes detected by RNA-Seq after exposure to IFNγ. Differentially expressed genes were sorted by fold change (Log_2_Fold Change). Adjusted *p* value is shown in the third column.

## Discussion

The results of our studies revealed a number of novel and intriguing findings that may help to advance the development of strategies for neuroprotection and/or repair.

Because of the inaccessibility of cells of interest in MS and the long course of disease development, creating platforms to study human cells derived from different patients will be critical to these advances. In order to generate a relatively high throughput system that allows intensive investigation of these cells, culture systems have been established, and initial results have reported the feasibility of differentiation into functional OLs from iPSCs. Both intermediate OPCs and mature OLs were generated from people with MS as well as from healthy controls. Importantly, in addition to the demonstration of an OL phenotype, the cells were shown to successfully myelinate a shiverer mouse, an animal model that cannot form compact myelin due to MBP deficiency [20]. Consistent with these reports, our data also showed that iPSCs from people with either stable/low disability or progressive/high disability MS differentiated into OPCs and OLs, expressing canonical markers that span the timeline of development and ultimately producing MBP and myelin that wrapped axons.

OPCs have been identified at the site of MS lesions, and the apparent ability of progenitors to mature contributes to the great remaining mystery of why the process of remyelination fails, and as a corollary why treatments to promote remyelination have not achieved greater success in human trials [46] despite promising results in animal modeling [21, 47]. One critical factor that is often not considered in the drug screening process or in *in vivo* models is the diseased environment. In people with MS, the local milieu includes secreted cytokines by invading cells as well as by resident affected cells that alters the balance of anti- and pro-inflammatory signals, growth factors and debris clearance [48, 49]. For example, recent studies have shown that iPSC-derived neural progenitor cells (NPC), from people with primary progressive MS (PPMS) did not provide adequate trophic or protective support for rat OPCs in culture [50], indicating that the environment in which remyelination is occurring in people may be complex. Thus, modeling this environment in culture may provide additional information regarding remyelination. Additional work from the same group later showed that diseased NPCs highly upregulated senescence genes, with HMGB-1 as a potential culprit in the failure of diseased NPCs to support rat OPC growth, from both local and migrating cells [51].

In addition to the absence of trophic effects, the disease environment is also challenged by the presence of inflammation, which is a prominent feature in early-stage MS. An array of proinflammatory cytokines have been implicated, including IL-17, IL-22, IL-1, IL-12, tumor necrosis factor-α, and IFNγ [52]. IFNγ is increasingly recognized as a driving factor in the pathology for its ability to amplify an inflammatory response by orchestrating the upregulation of CCL5, MIF, and CCL27 cytokines via activation of astrocytes and T-cells [53]. The effects also inhibit OPC differentiation and remyelination, in part because IFNγ foments a hostile environment that is not conducive to regeneration by inducing cell death (at high concentrations) i.e. STAT1/IRF-1 pathway [24], inhibiting cell cycle exit [27], and inflicting endoplasmic reticulum stress [29].

To mimic an inflammatory disease-type environment, we sought to investigate effects of chronic exposure of low-dose IFNγ on our cultures. In a long-term culture setting, a very low-dose was sufficient to dramatically inhibit the maturation of OLs, and the earlier the phase of culture and the longer the cells were exposed, the greater the effect. Our studies are consistent with previous findings in that IFNγ has a milder effect on immature rodent OLs, and a minimal effect on mature OLs [26, 54]. The mechanism behind this difference remains unclear, but highlights a critical vulnerable stage of development. Consistent with previous studies demonstrating that low-dose IFNγ did not promote apoptosis [27], our data also indicated no difference in cell death between untreated and interferon groups, which suggests that at least in low levels of inflammation, killing of the OPCs may also not be the primary cause of failure to remyelinate. It is interesting to speculate that the profound inhibitory effect that IFNγ exerts on OPC differentiation could contribute to the failure to remyelinate in a disease setting. The presence of OPCs at the sites of injury [8, 9] and evidence of early remyelination in MS [7, 55] strongly suggest that the capability to replace OLs and repair myelin is present. One possibility thus is that OPCS do migrate properly to site of injury to begin the repair process, but ultimately are halted in the differentiation process by the presence of a pro-inflammatory environment, and the effects of even low-dose cytokine could be sufficient to effectively shut down the repair process, in theory. IFNγ is a potent and pleiotropic cytokine that has been shown to affect mitochondrial function, which is considered a cause of axonal degeneration that accompanies failed remyelination [56, 57]. Interferon also induces oxidative stress, which in turn can cause DNA damage [58]. A significant implication of these data is that screening strategies to discover agents to promote endogenous differentiation may benefit from considering their effects under chronic low-grade inflammation, since therapies that promote OPC differentiation and/or survival in a homeostatic state may not be as effective in the presence of inflammatory molecules, and testing in the presence of such an environment may help to identify more potent and effective agents.

It is becoming clearer that progenitors of most cell types are not unalterably committed to a linear and unwavering path but rather may be influenced by environmental conditions, including growth factors and cytokines. In support of this notion, for example, previous work showed that IFNγ inhibited differentiation of rodent A2B5+ progenitor cells, reducing the number of mature O1+ oligodendrocytes, while enhancing proliferation of immature OPCs [27]. Our data is consistent with the suppression of maturation, but in our long-term culture of human cells, there was neither retention nor selective proliferation of early progenitors overall, but rather, the acquisition of a neuronal phenotype in at least a portion of cells. This finding parallels a previous discovery in microglia, in which exposure to a low-dose of IFNγ led to the acquisition of a neuronal-like phenotype [59]. Consistent with these findings, another report showed *in vitro* that IFNγ induced neuronal differentiation of neural stem cells in a dose-dependent manner. Together, these data support the notion that IFNγ inhibits the development of oligo lineage cells while promoting the differentiation into a neuronal lineage [60]. This prevalent plasticity in various cell types in response to the environment highlights a critical point in the ability of cells to adapt to changing circumstances and local influences and possibly serve different roles under conditions of disease or infection, for example. One hypothetical consequence of such plasticity is that as cells convert to different phenotypes, the availability of functional cells needed to repair could be depleted. If the OPCs and progenitors follow a different lineage pathways, then they would be unavailable to become OLs in this setting, for example. While numerous lines of evidence indicate that progenitor cells are able to acquire different characteristics, the mechanisms behind the redirection are less clear. As a means to begin to gain a greater understanding of the transcriptional basis for reprogramming in people, we undertook a transcriptomics analysis to identify genetic patterns of expression in a homeostatic and IFNγ-exposed states. The baseline genetic profile confirmed the identity of the PDGFRα sorted cells as OPCs. Interestingly, IFNγ exposure led to the differential expression of a number of genes in diverse pathways. Consistent with our observed effects on decreased myelin production, our screen identified an upregulation of one gene of interest, DAAM2 (Dishevelled Associated Activator of Morphogenesis 2), a Wnt signaling downstream effector. DAAM2 suppressed remyelination after white matter injury in rodents and humans [45], suggesting that this pathway may be a possible candidate for genes that could theoretically contribute to the observed effect. Another study demonstrated a correlation between decreased DAAM2 expression and improvements in remyelination in Guillain- Barre syndrome [61], suggesting that IFNγ may activate specific pathways redirecting the process. DAAM2 is also thought to be important for dorsal patterning in the development of the spinal cord [62], which is also consistent with these findings. Further investigation into the significance of this pathway in this setting would be necessary to parse out its function, however, but it is interesting to speculate that activation of specific programs of gene expression by cytokine exposure could dramatically alter the cells.

An intriguing re-direction of OPC function into an immune/inflammatory realm has been recently identified as well. Specifically, rodent OPCs exposed to IFNγ acquired properties typically associated with the immune system. Termed “inflammatory OPCs” these IFNγ–exposed cells upregulated molecules such as MHCs and further took on antigen presenting cell characteristics [63, 64]. The implications of this process are profound, in that it suggests the possibility that OPCs at the site of a lesion could convert to an immune-like cell that stimulates cytotoxic T cells and perpetuates a damaging immune response, while similarly converting away from a cell type designed to repair damage. Our transcriptomics analysis demonstrated this conversion on at least the RNA level in all of the samples we tested. Our present data showed upregulation of interferon response genes such as STAT1, IRF1, IRF2, IRF7, and IRF9, all of which could contribute to the perpetuation of an inflammatory phenotype. Aligning with the possibility of acquisition of antigen presenting cell characteristics, IFNγ also led to upregulation of peptide processing genes of importance to the immune system, including TAP1, TAP2, and ERAP1, along with the immunoproteasome genes PSME1, PSME2, PSMB8, and PSMB9. Further, MHC molecules necessary for antigen presentation including class I HLA-A and HLB-B, and class II CIITA were all highly upregulated in response to IFNγ. These results have intriguing implications in a disease setting in that it could be a means by which OPCs inadvertently worsen disease at the site at which repair is needed.

Gaining a greater understanding of OPC and OL function in people with MS is of paramount importance to uncovering mechanisms of disease and developing new targeted therapeutics. Taken together, our data demonstrate that in a nurturing environment, iPSCs derived from people with stable and progressive MS are capable of differentiating into mature OLs, but that this process is profoundly inhibited by chronic exposure to low-dose IFNγ. Our data have significant clinical relevance as they suggest that the environment in which differentiation therapies are administered may profoundly affect the efficacy of the treatment, and ultimately, a combination approach of decreasing inflammation in the CNS may be necessary for OPC differentiating/remyelinating agents to reach their full efficacy.
